# Infektionen des Rückenmarks und der angrenzenden Strukturen

**DOI:** 10.1007/s00115-023-01439-x

**Published:** 2023-02-23

**Authors:** Bettina Pfausler, Verena Rass, Anna Lindner

**Affiliations:** Universitätsklinik für Neurologie, Medizinische Universität Innsbruck, Anichstr. 35, 6020 Innsbruck, Österreich

**Keywords:** Myelitis, Spondylodiszitis, Poliomyelitis, Pathogene, Topografie, Myelitis, Spondylodiscitis, Poliomyelitis, Pathogens, Topography

## Abstract

Eine Entzündung des Myelons und der angrenzenden Strukturen kann durch Viren, Bakterien, Pilze und Parasiten verursacht werden. Während Viren bevorzugt das Myelon und die Radizes direkt infizieren oder eine sekundäre Immunantwort triggern, neigen Bakterien, Pilze und Parasiten zur Bildung von Abszessen, Granulomen und Zysten und können wie destruierende Osteomyelitiden zu einer sekundären Myelonkompression führen. Die ätiologische Eingrenzung eines akuten/subakuten spinalen Prozesses erfolgt anhand der klinischen Präsentation, der zeitlichen Dynamik der Symptomentwicklung, des Immunstatus, der Bildgebung und mikrobieller/molekularbiologischer Untersuchungen von Liquor und Serum. Aufgrund des Tropismus einzelner Erreger zu bestimmten Faserstrukturen und Zellverbänden im Rückenmark kann in Zusammenschau mit der Klinik, der Bildgebung und der Expositionsanamnese oft bereits zeitnah eine fokussierte Abklärung und Diagnose erfolgen. In diesem Artikel wird auf wichtige Erreger einer spinalen/paraspinalen Infektion, deren geografisches Vorkommen und die klinische und bildgebende Präsentation unter besonderer Berücksichtigung der anatomisch-topografischen Lokalisation und aktueller epidemiologischer Entwicklungen eingegangen. Der Ausbruch von Poliomyelitiserkrankungen durch zirkulierende Impfstoffpolioviren (cVDPV) sei hier im Speziellen erwähnt.

## Abstract (en)

Inflammation of the spinal cord and the adjacent structures can be caused by viruses, bacteria, fungi and parasites. Viruses predominantly infect the spinal cord and the nerve roots directly or trigger a secondary immune response, whereas bacteria, fungi and parasites tend to form abscesses, granulomas and cysts and can lead to a secondary compression of the spinal cord, similar to a destructive osteomyelitis. The etiological clarification of an acute or subacute spinal process is carried out based on the clinical presentation, the time course of the development of symptoms, the immune status, neuroimaging and microbial and/or molecular biological examinations of cerebrospinal fluid and serum. The tropism of individual pathogens to certain fiber structures and cellular clusters in the spinal cord in synopsis with the clinical presentation, neuroimaging and a history of exposure, can often quickly lead to a focused clarification and diagnosis. This article deals with important pathogens of spinal and paraspinal infections, the geographical distribution, the clinical and neuroimaging presentation with special consideration of the anatomical and topographical localization and recent epidemiological developments. Particular attention is paid to the outbreak of poliomyelitis due to circulating vaccine-derived poliovirus (cVDPV).

## Hintergrund

Akute Erkrankungen des Rückenmarks (RM) werden als Myelopathie bezeichnet und haben eine sehr heterogene Ursache [31]. Eine Myelitis im engeren Sinn ist eine Entzündung des RM durch Viren, Bakterien, Parasiten und Pilze. Das Pathogen kann dabei direkt das RM affizieren, eine Immunreaktion triggern (post-/parainfektiöse Myelitis) oder durch einschmelzende destruierende Prozesse paraspinaler Strukturen zu einer Myelonkompression führen. Epidemiologische Zahlen zu spinalen Infektionen sind dürftig, auch gibt es abhängig vom Pathogen eine große geografische Varianz (Tab. 1).

**Table 1:** 

		Endemische Region	Wichtige klinische Merkmale
Picornaviren	Polio-Wildvirus Typ 1^a,b^	Pakistan, Afghanistan, Mosambik, Malawi	AFM (asymmetrisch) Motorische Hirnnervenkerne Meningitis, Enzephalitis
	cVDPV	Weltweit möglich	
	Enterovirus-D68^b^	Weltweit	AFM (asymmetrisch) Motorische Hirnnervenkerne Meningitis, Enzephalitis
	Enterovirus-A71^b,c^	Weltweit	AFM (asymmetrisch) Motorische Hirnnervenkerne Meningitis, Enzephalitis
	Hepatitis-A-Virus^d^	Weltweit	Transverse Myelitis (selten)
Flaviviren	Frühsommermeningoenzephalitisvirus (FSME)	Europa, Russland, China	AFM (5–15 %) Meningitis, Enzephalitis
	West-Nil-Virus	Nordamerika, Mittel‑, Südeuropa, Subsahara-Afrika, Australien, Neuseeland	Myelitis, AFM Meningoenzephalitis
	Dengue-Virus	Zentral- und Südamerika, Subsahara-Afrika, Süd- und Südostasien	Myelitis selten Enzephalitis, Myelitis
	Japanische-Enzephalitis-Virus	China, Süd- und Südostasien	Myelitis, AFM Enzephalitis
	St.-Louis-Enzephalitis-Virus	Nord- und Südamerika	Meningoenzephalitis, AFM
	Zika-Virus	weltweit	Guillain-Barré-Syndrom Myelitis
	Hepatitis-C-Virus^d^	Weltweit	Guillain-Barré-Syndrom
Herpesviren	Herpes-simplex-Virus 1	Weltweit	Enzephalitis, selten Myelitis, Myeloradikulitis
	Herpes-simplex-Virus 2	Weltweit	Kauditis (Elsberg-Syndrom) Radikulomyelitis Rezidivierende Meningitis
	Varicella-zoster-Virus	Weltweit	Radikulitis Myelitis bei Immunsuppression
	Epstein-Barr-Virus	Weltweit	LETM (parainfektiös) Meningoenzephaloradikulitis
	Zytomegalievirus	Weltweit	Polyradikulitis, Myelitis Enzephalitis (nur bei Immunsuppression)
	Humanes Herpesvirus 6/7	Weltweit	Myelitis, Enzephalitis (nach allogener Stammzelltransplantation)
Retroviren	Humanes Immundefizienzvirus	Weltweit	Chronische Myelitis Meningoenzephalitis akut
	Humanes T‑Zell-lymphotropes Virus (HTLV-1)	Japan, Subsahara-Afrika, Zentral- und Südamerika, Karibik	Chronische Myelitis (tropische spastische Paraparese)
Paramyxoviren	Mumpsvirus	Weltweit	Meningitis, Meningoenzephalitis, Myelitis selten
	Masernvirus	Weltweit Lokale Epidemien	Exanthem, Enzephalitis, Myelitis
Rhabdoviren	Rabiesvirus	Weltweit	AFM (paralytischer Verlauf) bei 20 % Enzephalitis

*AFM* „acute flaccid paralysis/akute schlaffe Lähmung“, *cVDPV* „circulating vaccine derived poliovirus“, *LETM* longitudinale extensive transverse Myelitis

^a^Polio-Wildvirus Typ 2 und 3 eradiziert

^b^Vorwiegend bei Kindern, Übertragung orofäkal durch Tröpfchen und Schmierinfektionen

^c^Verursacher der Hand-Fuß-Mund-Erkrankung und von Herpangina

^d^Hepatitisviren gehören unterschiedlichen Virusfamilien an

## Terminologie

Anhand der anatomisch-topografischen Präsentation und der Bildgebung kann zwischen einer einfachen transversen Myelitis (TM) – nur über einzelne Segmente – einer longitudinalen extensiven transversen Myelitis (LETM) – die Läsionslast erstreckt sich über mehr als 3 Segmente –, einer Radikulitis und Radikulomyelitis differenziert werden. Wie radiologische Beschreibungen (TM, LETM) kann die zeitliche Dynamik der Entwicklung der Symptome, Begleitsymptome, Immunstatus und Reise- und Risikoverhalten in der Einengung der Ätiologie hilfreich sein [2, 20].

## Diagnostisches Vorgehen

Das diagnostische Vorgehen umfasst die klinische Untersuchung, die Bildgebung und mikrobiologische/serologische Untersuchungen. Eine Abklärung hinsichtlich einer nichtinfektiösen Ursachen einer Myelopathie – immunologische/paraneoplastische Erkrankungen, vaskuläre oder toxisch-metabolische Prozesse – muss anhand von klinischer Präsentation und Bildgebung getroffen werden.

### Klinik

Eine infektiöse Myelitis tritt subakut über Stunden bis Tage auf, chronische Myelitiden mit Symptomentwicklung über Monate und Jahre sind in unseren Breiten eine Rarität und auf Erreger wie das humane T‑Zell-lymphotrope Virus‑1 (HTLV-1) oder das humane Immundefizienzvirus (HIV) begrenzt.

Aufgrund der Anordnung der Faserstrukturen im RM können die Symptome partiell ausgeprägt sein

Kernsymptom der spinalen Erkrankung ist die Querschnittssymptomatik mit – abhängig von der Läsionshöhe – einer Para‑/Tetraparese, einem sensiblen Niveau, Blasen‑, Mastdarm- und autonome Störungen. Aufgrund der topografischen Anordnung der Faserstrukturen im RM und der nicht immer symmetrischen Affektion können die Symptome partiell ausgeprägt sein. Während das akute spinale Trauma im sog. spinalen Schock einen schlaffen Muskeltonus, eine Areflexie und fehlende Babinski-Zeichen hat, zeigt sich bei der sich subakut entwickelnden Myelitis eine spastische Muskeltonuserhöhung und pathologische Reflexe [27]. Standardisierte Untersuchungsprotokolle, wie sie für das spinale Trauma in Verwendung sind, können auch für die Beurteilung einer Myelitis verwendet werden [3].

### Bildgebung

Die Magnetresonanztomographie (MRT) ist die zu bevorzugende Untersuchung zur Abklärung einer spinalen Symptomatik [32]. Bei Kontraindikation zum MRT (ältere Herzschrittmacher und orthopädische/traumatologische Prothesen) müssen raumfordernde Prozesse, welche zu einer Myelonkompression führen, mittels Computertomographie ausgeschlossen werden.

Beurteilt werden Ausdehnung des Prozesses, die schwerpunktmäßige Affektion bestimmter anatomischer Strukturen und eine meningeale und radikuläre Mitbeteiligung. Eine Kontrastmittel(KM)-Aufnahme kann insbesondere am Beginn einer Erkrankung auch fehlen. Eine Positronenemissionstomographie (PET) wird routinemäßig in der Abklärung der akuten Myelitis nicht eingesetzt und bleibt auf Einzelfallbeschreibungen limitiert [19].

### Laboruntersuchungen

Die Basisuntersuchungen des Liquors umfassen Zellzahl, Protein, Liquor‑/Serumglukose-Ratio und Immunglobulin(Ig)G-Index. Molekularbiologische Techniken wie die Multiplex-PCR(„polymerase chain reaction“) haben in den letzten Jahren die Abklärung für Infektionen entscheidend verbessert, dennoch gelingt es nicht immer, ein Agens zu isolieren. Die erregerfokussierte Aufarbeitung des Liquors erfolgt entsprechend den Empfehlungen zur Abklärung einer Meningitis/Enzephalitis [15, 22].

## Empirische Therapieentscheidungen

Die initial meist empirische Therapieentscheidung – virostatische vs. antimikrobielle Therapie – erfolgt anhand der Anamnese, Klinik, Bildgebung und der gängigen Infektparameter. Eine Leukozytose, eine Linksverschiebung im Differenzialblutbild, erhöhtes C‑reaktives Protein, Prokalzitonin und Interleukin sprechen für ein bakterielles Geschehen. Fehlende oder eine nur minimale Auslenkung der Entzündungsparameter können unter einer Therapie mit Immunbiologika auftreten, sodass dies bei der Interpretation der Entzündungsparameter berücksichtigt werden muss [1]. Die Wahl des Antibiotikums/Virostatikums erfolgt entsprechend der bakteriellen oder viralen ZNS-Infektion [15, 22]. Bei einer (Poly‑)Radikulitis und Aufenthalt in einem Zeckenendemiegebiet oder nach Zeckenstich (Borreliose) ist eine intravenöse Monotherapie mit Ceftriaxon, bei segmentalen Hauteffloreszenzen (Varicella-zoster-Infektion) eine Monotherapie mit Aciclovir/Valaciclovir vertretbar [25]. Die Indikation einer gegebenenfalls zusätzlichen Kortikosteroidtherapie, insbesondere bei langstreckigen Veränderungen oder Begleitödem bei Abszessformationen, muss individuell entschieden werden.

## Virale Myelitis

Virale Infektionen des zentralen Nervensystems (ZNS) sind deutlich häufiger als Infektionen durch Bakterien, Pilze oder Protozoen (Tab. 1). Die Invasion ins ZNS erfolgt hämatogen während einer Virämie, aber auch axonal retrograd entlang peripherer Nerven. Bestimmte Viren zeigen einen unterschiedlichen Tropismus für Faserstrukturen und Zellverbände im RM [33].

### Enteroviren

Enteroviren (EV) sind ubiquitär vorkommend und verursachen vorwiegend bei Kindern respiratorische und gastrointestinale Infekte. Ein myelitischer Verlauf ist bei allen EV-Infektionen möglich, wenngleich selten.

Das Poliovirus (PV) ist das klassische Virus für eine motorische Vorderhornzellerkrankung (Kinderlähmung). Seit 2012 kommt es weltweit zu clusterartigem Auftreten polioähnlicher Erkrankungen durch Nonpolioviren („acute flaccid paralysis/myelitis“, AFP/AFM). Non-PV-Erkrankungen stellen eine eigene Krankheitsentität dar und unterliegen speziellen Surveillance-Programmen [7, 23].

#### Poliovirus

Der Mensch ist das einzige Reservoir für PV. Seit 1988 wurde von der World Health Organization (WHO) ein weltweites Poliovireneradikationsprogramm gestartet und erfolgreich umgesetzt (WHO Global Polio Eradication Initiative). Das Polio-Wildtypvirus (PWT) Typ 1 gibt es noch in Pakistan, Afghanistan, Mozambik und Malawi, PWT-Typ 2 und 3 gelten als eradiziert. Aufsehen erregten zuletzt Erkrankungen mit zirkulierenden Impfstoffpolioviren („circulating vaccine derived poliovirus“, cVDPV) in New York im Sommer 2022, in Jerusalem im Frühjahr 2022 sowie in der Ukraine 2021 [11].

Bei Erkrankung ist eine strikte Isolation einzuhalten, eine spezifische Therapie gibt es nicht

Nur 5 % der PV-Infektionen verlaufen symptomatisch. Die schwere spinale und/oder bulbäre paralytische Verlaufsform tritt bei weniger als 1 % auf. Die Paresen sind initial von heftigen Muskelschmerzen, Muskelspasmen und fallweise lokalisierten Sensibilitätsstörungen begleitet [28]. Das Virus kann aus Rachensekret und Stuhl isoliert werde. Die Ausscheidung im Stuhl kann über mehrere Wochen persistieren. Bei einer Erkrankung ist eine strikte Isolation einzuhalten, eine spezifische Therapie gibt es nicht. Auch Jahre bis Jahrzehnte nach der Erkrankung kann es zur Entwicklung eines Postpoliosyndroms mit fortschreitenden Paresen und Atrophien in den ursprünglich betroffenen Extremitäten kommen [21].

#### Enterovirus D-68 und Enterovirus A-71

Ein Zusammenhang zwischen einer Enterovirus(EV)-D-68- und EV-A-71-Infektion mit einer AFM wird immer wieder suszipiert. EV D-68 verursacht in der Regel einen unkomplizierten respiratorischen Infekt, EV A-71 führt zur Hand-Fuß-Mund-Erkrankung und zur Herpangina. Einige Tage nach dem primären Infekt kommt es zur Ausbildung einer asymmetrisch schlaffen Lähmung insbesondere der oberen Extremitäten. Ein Nachweis der Viren im Liquor gelingt kaum. Das MRT zeigt die typischen Veränderungen am motorischen Vorderhorn. Therapieversuche mit Kortikosteroiden, Immunglobulinen und Plasmapherese werden immer wieder berichtet [5].

### Flaviviren

Zu den für den Neurologen wichtigen Flaviviren zählen das West-Nil-Virus (WNV), das St.-Louis-Enzephalitis-Virus, das japanische Enzephalitisvirus, die Dengue-Viren, das Zika-Virus und das Frühsommermeningoenzephalitisvirus (FSMEV). Flaviviren werden durch Arthropoden (Moskitos, Zecken) übertragen. Erkrankungen manifestieren sich hauptsächlich als Meningitis und Meningoenzephalitis. Aufgrund des Tropismus der Viren zur grauen Substanz des RM und der Hirnnervenkerne müssen sie jedoch bei entsprechender Expositionsanamnese auch in der Abklärung der AFM miteinbezogen werden.

#### Frühsommermeningoenzephalitisvirus

**Figure 1:**
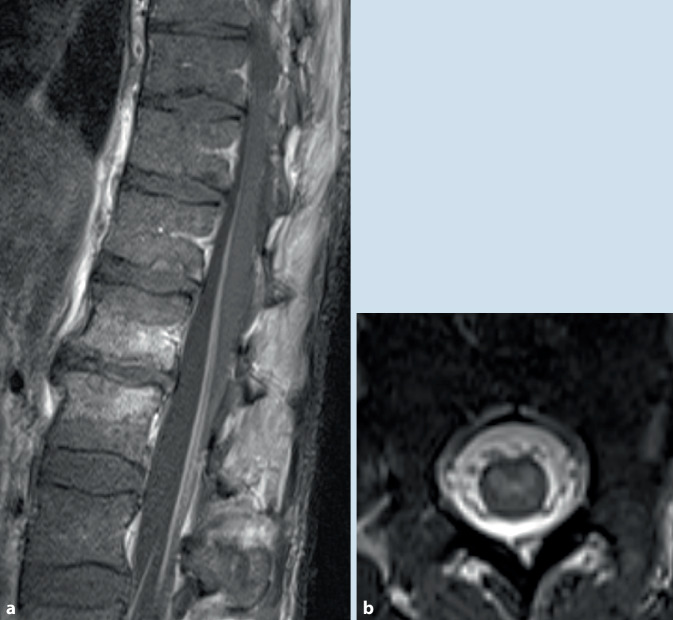

Das FSMEV ist in Europa endemisch und wird durch den gemeinen Holzbock (*Ixodes ricinus*) übertragen. Das Virus ist in der Speicheldrüse der Zecke, die Übertagung erfolgt somit unmittelbar mit dem Stich der Zecke. Eine FSME-Erkrankung verläuft klassischerweise biphasisch. Eine „Polio-like“-Symptomatik mit Befall der motorischen Vorderhornzellen wird in 5–15 % der Fälle gesehen und hat eine hohe Morbidität (Abb. 1). Die Diagnose erfolgt über den Nachweis spezifischer Antikörper in Serum und Liquor, die PCR-Diagnostik hat keine klinische Alltagsrelevanz. Die FSME zählt zu den impfpräventablen Erkrankungen, es gibt keine spezifische Therapie [29].

#### West-Nil-Virus

Das WNV ist auch in Europa zwischenzeitlich angekommen [8]. Das WNV hat eine geringe neuroinvasive Potenz, myelitische „Polio-like“-Verläufe treten in weniger als 1 % auf [4]. Die Diagnose erfolgt über serologische Untersuchungen, eine spezifische Therapie gibt es nicht.

#### Japanisches Enzephalitisvirus, Dengue-Viren, Zika-Virus, St-Louis-Enzephalitis-Virus

Diese Viren haben in Europa eine untergeordnete Bedeutung. Das japanische Enzephalitisvirus ist die häufigste Ursache einer AFM bei Kindern in Vietnam. 2015 kam es ausgehend von Südamerika zu einer Pandemie mit dem Zika-Virus. In Folge kam es zu einem gehäuften Auftreten eines Guillain-Barre-Syndroms und einer Mikrozephalie. Das St-Louis-Enzephalitis-Virus ist bei einer AFM differenzialdiagnostisch in jenen Regionen miteinzubeziehen, in welcher das Virus endemisch vorkommt [12].

### Herpesviren

Herpesviren persistieren nach einer primären Infektion asymptomatisch in sensiblen Ganglien, Endothelzellen und Lymphozyten und können von dort reaktiviert werden. Primäre Infektionen sind im Kleinkindesalter möglich.

#### Herpes-simplex-Virus Typ 1 und 2

Während Herpes-simplex-Virus Typ 1 (HSV1) vorwiegend eine Enzephalitis verursacht, ist die HSV2-Erkrankung beim Erwachsenen mit einer Myeloradikulitis assoziiert. Eine besondere Manifestation ist das Elsberg-Syndrom, bei dem es zu einer Affektion der lumbosakralen Radizes und des Conus medullaris kommt [30].

#### Varicella-zoster-Virus

Häufigste Manifestation einer Varicella-zoster-Virus(VZV)-Reaktivierung ist der dermale Herpes zoster. Eine Myelitis tritt überwiegend bei immunsupprimierten Personen auf. Eine zeitgleiche Präsenz von Hauteffloreszenzen ist nicht obligat [24]. Bei Risikogruppen und älteren Menschen wird heute eine Impfung empfohlen.

#### Zytomegalievirus

Eine Zytomegalievirusinfektion tritt nur bei schwerer Immunsuppression auf und kann zu einer schmerzhaften Polyradikulitis oder einer LETM führen. Das MRT ist insbesondere bei der polyradikulitischen Präsentation meist negativ.

#### Epstein-Barr-Virus

Verschiedene neurologische Erkrankungen werden immer wieder mit Ebstein-Barr-Virus-Infektionen in Zusammenhang gebracht. Eine LETM oder Polyradikulitis kann Wochen nach einer infektiösen Mononukleose auftreten. Das EBV ist somit vermutlich nicht primäres neurotropes Agens, sondern Trigger eines immunmediierten parainfektiösen Geschehens [18].

#### Humanes Herpesvirus 6 und humanes Herpesvirus 7

Nach einer allogenen Stammzelltransplantation kommt es häufig zu asymptomatischen Reaktivierungen von HHV(humanes Herpesvirus)-6 und -7. Myelitiden und Enzephalitiden werden dabei nur als seltene Komplikationen beschrieben [13].

#### Humanes T-Zell-lymphotropes Virus 1, humanes Immundefizienzvirus

Humanes T‑Zell-lymphotropes Virus 1 (HTLV-1) und humanes Immundefizienzvirus (HIV) verursachen eine langsam progrediente chronische Myelitis mit Affektion der Hinter- und Seitenstränge. Erkrankte entwickeln über Jahre eine sensorische Ataxie, spastische Paresen und Blasenstörungen [17]. Die Erkrankung ist eine Ausschlussdiagnose.

### „Severe acute respiratory syndrome coronavirus type 2“

Seit dem Ausbruch der COVID-19(„coronavirus disease 2019“)-Pandemie 2019 sind mehr als 300.000 Publikationen entstanden. Verschiedenste neurologische Symptome werden im Zusammenhang mit dem Virus gesehen. Radikulitiden und Myelitiden traten sowohl mit einer akuten Erkrankung als auch nach Impfungen auf [9].

## Pilze und Parasiten

Pilze und Parasiten sind seltene Ursachen einer spinalen Infektion. Pilze bilden Granulome, destruierende Osteomyelitiden und intramedulläre Abszesse und führen damit zu einer RM-Kompression. Auch direkte Gefäßinfiltration mit spinalen Infarkten sind möglich.

*Schistosoma mansonii* ist die häufigste Ursache einer akuten lumbalen Myelopathie in Südamerika

Auch Parasiten bilden das Myelon bedrängende zystische Formationen und Granulome, aber auch eine LETM oder eine Radikulitis sind beschrieben (Tab. 2). *Schistosoma mansonii* ist die häufigste Ursache einer akuten vorwiegend lumbalen Myelopathie bei jungen Erwachsenen in Südamerika. Bei einer Eosinophilie im Blut ist bei symptomatischen Reiserückkehrern und Migranten immer eine parasitäre Erkrankung auszuschließen.

**Table 2:** 

		Endemische Region	Wichtige klinische Merkmale
Bandwürmer (Zestoden)	*Taenia solium*^a^ (Neurozystizerkose)	Zentral- und Südamerika, Subsahara-Afrika, Süd- und Südostasien	(Verkalkte) Zysten, Granulome, selten im Myelon
	*Echinococcus spp.*	Mitteleuropa, USA, Zentralasien, Sibirien, Ostafrika, China, Japan	Zerebrale und spinale Zysten
Spulwürmer^b^ (Nematoden)	*Toxocara canis*	Weltweit	Meningoenzephalitis, Myelitis, Radikulitis, Hirnnervenausfällen Vaskulitis
	*Gnathostoma spinigerum*	Südostasien	Radikulitis, (hämorrhagische) Myelitis
	*Angiostrongylus* *cantonensis*	Südostasien	Radikulitis, Myelitis, Granulome
	*Angiostrongylus* *costaricensis*	Zentralamerika	Radikulitis, Myelitis, Granulome
Saugwürmer (Trematoden)	*Schistosoma mansoni* *Schistosoma haematobium*	Zentral- und Südamerika, Subsahara-Afrika	LETM, Granulome
	*Paragonimus westermani*	Asien, Afrika, Südamerika	Zerebrale und spinale Zysten
Protozoen	*Toxoplasma gondii*	weltweit	Abszess, Enzephalitis, selten spinale Abszesse

^a^Die Neurozystizerkose wird durch die Larve von *Taenia solium* – Cysticercus cellulosae – verursacht

^b^Die Larven invadieren Radizes und Myelon (eosinophile Radikulomyelitis) und Subarachnoidalraum

## Bakterielle Infektionen des Rückenmarks und Spondylodiszitis

**Figure 2:**
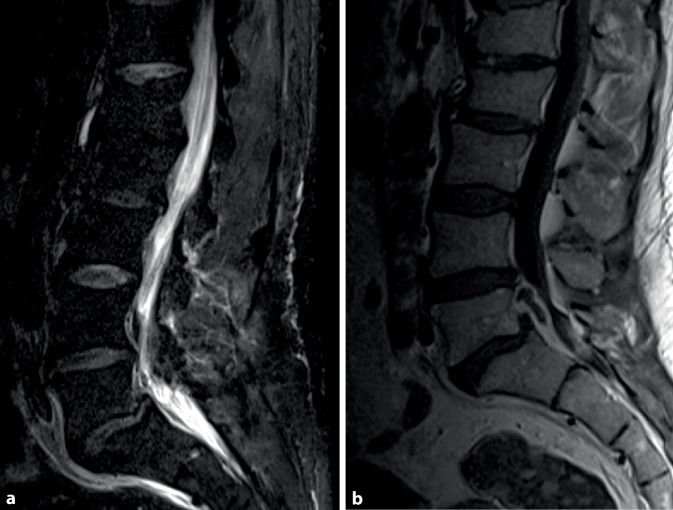

Pyogene Infektionen des RM sind heterogene Erkrankungsbilder. Isolierte intraspinale Abszesse sind selten. Häufiger sind Infektionen der Diszi und der Wirbelkörper (Spondylodiszitis). Von dort kann sich die Infektion nach epidural und paravertebral ausbreiten und zu einer sekundären Myelonkompression und Plexopathie führen (Abb. 2).

### Spondylodiszitis und epiduraler Abszess

In den letzten Jahren wird eine Zunahme der spinalen spondylitischen Infektionen beobachtet. Dies dürfte einerseits durch die Risikofaktoren für eine Infektion – höheres Alter, Komorbiditäten, Immunsuppression, intravenöser Drogenmissbrauch und Infiltrationsbehandlung, Instrumentation der Wirbelsäule –, anderseits aber auch durch die bessere bildgebende Diagnostik bedingt sein.

Häufigster Erreger einer Spondylodiszitis ist *Mycobacterium tuberculosis*

Symptome einer Spondylodiszitis (SD) sind progrediente therapierefraktäre Rückenschmerzen, radikuläre Schmerzausstrahlung auf Höhe des affizierten Segments, subfebrile Temperaturen und systemische Entzündungszeichen. Häufigster Erreger einer SD in unseren Breiten sind Staphylokokken, weltweit und historisch das *Mycobacterium tuberculosis*. Bei ausgedehntem epiduralem Abszess, neurologischer Begleitsymptomatik oder Instabilität der Wirbelsäule ist neben der immer notwendigen antibiotischen Therapie die chirurgische Intervention obligat. Die Regredienz der Rückenschmerzen und fallende Entzündungswerte sind sensitive Verlaufsparameter einer erfolgreichen Therapie. Die Dauer der antibiotischen Therapie beträgt mindestens 6 Wochen [16].

#### *Mycobacterium tuberculosis* und *Brucella spp.*

Mehr als 2 Mrd. Menschen haben eine latente Tuberkulose (TB). Die tuberkulöse Spondylodiszitis (Pott-Abszess) ist die häufigste Manifestation einer ossären Tuberkulose. Weitere Manifestation einer spinalen Tuberkulose sind eine Arachnoiditis, Granulome und eine Meningoradikulitis [25]. Die Therapiedauer einer Neuro-TB beträgt mindestens 9 bis 12 Monate [26].

Die Brucellose ist die häufigste Zoonose weltweit. Brucellen kommen im Mittelmeerraum, Balkan, mittleren Osten und Südamerika vor. Eine Infektion erfolgt über Milch und -produkte infizierter Tiere, die Inkubationszeit beträgt 1 bis 2 Monate. Bei einer systemischen Erkrankung (Mittelmeerfieber) kann es zu einer Spondylitis, intraspinalen Abszessen und Granulomen kommen. Die Diagnose erfolgt durch Kultur auf speziellen Nährmedien, serologisch und mittels PCR-Testung. Aufgrund der hohen Seroprävalenz in Endemiegebieten und Kreuzreaktionen der PCR mit anderen Erregern ist die Diagnose diffizil [6].

### Spirochäten

*Borrelia spp. (B. burgdorferi, afzelii, garinii*) und *Treponema pallidum* (Syphilliserkrankung) sind die wichtigsten Vertreter einer neurologischen Manifestation einer Spirochäteninfektion.

#### Borrelien

Borrelien finden sich im Vormagen der Zecke (*Ixodes ricinus*). Die Infektion erfolgt erst nach Stunden, wenn die Zecke die Mahlzeit regurgitiert. Neben einem häufig inapperenten klinischen Verlauf kann es zu Hirnnervenlähmungen, einer schmerzhaften Oligoradikulitis (Bannwarth-Syndrom) und in seltenen Fällen einer transversen Myelitis kommen. Die Diagnose erfolgt in Zusammenschau der Klinik und der positiven Serologie im Liquor [25].

#### Syphillis

Die den letzten Jahren wird eine steigende Inzidenz der Lues, in Koinzidenz mit einer HIV-Infektion, berichtet [14]. Im frühen Stadium kann es durch eine Meningovaskulitis zu einer spinalen Manifestation kommen. Die vor der Möglichkeit der antibiotischen Therapie beschriebene Spätmanifestation einer Tabes dorsalis mit der Degeneration der Hinterstränge ist nur mehr historisch zu sehen. Dabei kam es Jahre nach einer unbehandelten Primärinfektion zu einer spastischen ataktischen Gangstörung und einschießenden lanzierenden Schmerzen [10]. Die Diagnose einer Lues erfolgt über verschieden serologische Testschritte und über eine PCR-Testung im Liquor. Kreuzreaktionen mit Borrelien sind möglich.

## Fazit für die Praxis

- Erregerassoziierte Infektionen des Rückenmarks sind deutlich seltener als jene des Gehirns.
- Die moderne Bildgebung erlaubt eine gute morphologische Beschreibung des Prozesses, darf aber nie der klinischen Untersuchung und Anamneseerhebung untergeordnet werden.
- Reisen und Migration erfordern Kenntnisse über die geografisch Verbreitung von Erregern und der breitere Einsatz von Immunsuppressiva konfrontiert auch mit ungewöhnlicheren Erregern und Manifestationen.
